# Sclerotherapy in orbital lymphangioma: A case report

**DOI:** 10.1016/j.radcr.2023.09.008

**Published:** 2023-10-03

**Authors:** Eppy Buchori Aristiady, Hilman, Harry Galuh Nugraha, Muhammad Dilga Caesario, Angga Kartiwa, Anne Susanty

**Affiliations:** a Radiology Department, Hasan Sadikin Hospital, Faculty of Medicine, Padjadjaran University, Bandung, Indonesia; b Opthalmology Department, National Eye Center, Cicendo Eye Hospital, Faculty of Medicine, Padjadjaran University, Bandung, Indonesia

**Keywords:** Orbital lymphangioma, Radiological Imaging, Sclerotherapy

## Abstract

Orbital lymphangioma is a rare benign lymphatic and vascular malformation, which is distinguished by its abnormal endothelial ducts and can cause proptosis in the patients. Radiological imaging is essential in confirming the diagnosis. Sclerotherapy is an alternative treatment option if surgery is not an option or is too risky. This report presents a rare case of a 4-year-old girl who had complained of bulging her right eye since she was 3 years old. Because surgery can be challenging to the surrounding vital orbital structure, the patient underwent several sclerotherapy sessions which yielded good results on the patient.

## Background

Lymphangioma is a localized multi-cystic malformation of the lymphatic and vascular systems that most commonly affects children' heads and necks. Lymphangioma accounts for 0.3%-4% of all orbital tumors and it is not considered hamartoma as the orbit does not typically contain lymphatic vessels [1]. Proptosis may develop gradually in some patients, especially children who were diagnosed after trauma or infection, although it may also occur spontaneously. The orbital lymphangioma may also enlarge due to systemic infection [2]. In children, early and effective treatment is crucial for preserving vision and preventing amblyopia [3]. The diagnosis of lymphangioma is best confirmed by radiological imaging to simultaneously assess its size and extent [3,4]. Lymphangioma often relates to vital orbital structures, therefore debulking or complete excision may be not possible as they can cause damage to nearby structures. Surgery also carries a significant risk of recurrence and scarring. In these situations, sclerotherapy is an alternative treatment and has a good success rate [5]. In this report, we present a rare case of a 4-year-old girl with orbital lymphangioma and discuss the role of sclerotherapy in the treatment of orbital lymphangioma.

## Case presentation

**Fig. 1:**
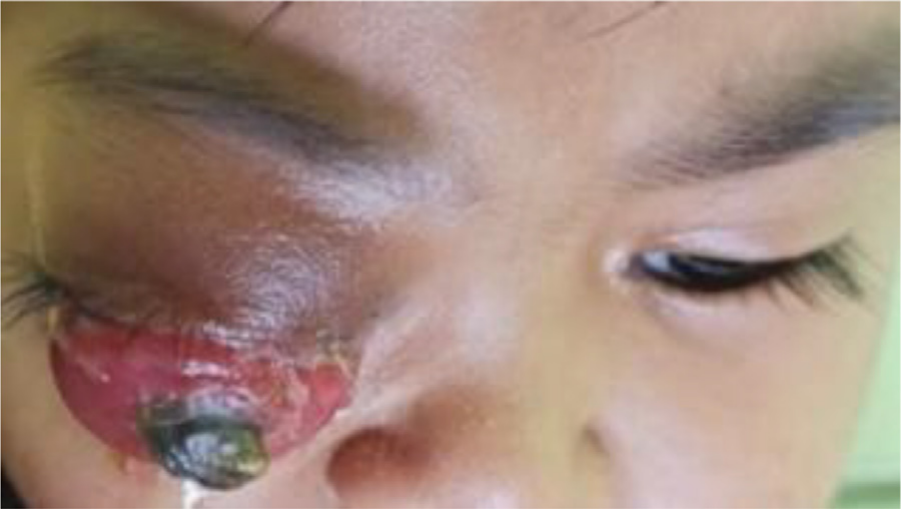
Extraocular mass on the right eye of the 4-year-old patient before sclerotherapy.

A 4-year-old girl came to our hospital with chief complaint of bulging right eye, which was often accompanied with yellowish pus discharge since she was 3 years old. The patient also experienced a dry cough, runny nose, decreased appetite, and weight loss. Upon examination, the patient was cranky, weak, and sleepy. On her right eye, there was a 3 × 2 × 2 cm ocular mass covered by conjunctiva (Fig. 1). The mass was immobile without any push or blood discharge and caused pain if palpated. Ophthalmology examination of the right eye revealed hyperlacrimation, discharge, conjunctival hyperemia, keratopathy, and no light perception.

**Fig. 2:**
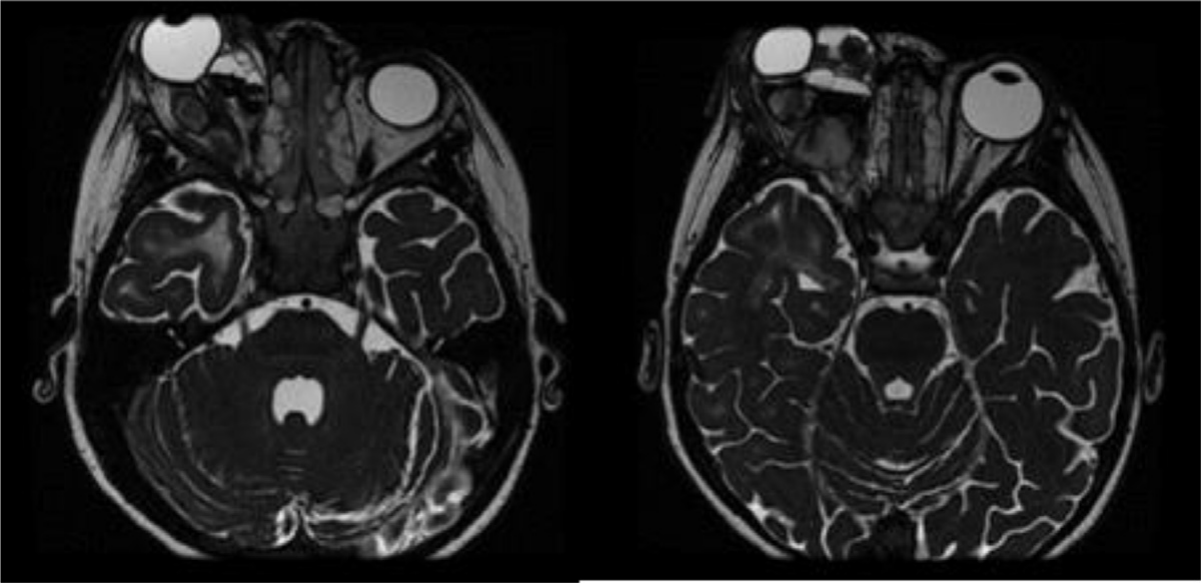
MRI presclerotherapy showed inhomogeneous lesion in right retrobulbar which infiltrated right intraconal and extraconal fat, right nerve optic, and right superior-medial-anterior recti. The distance between the anterior margin of the right ocular bulb and the right interzygomatic line was 2.36 cm.

Orbital MRI showed an inhomogeneous lesion in the right retrobulbar that infiltrated the right intraconal and extraconal fat, right nerve optic, and right superior-medial-anterior recti; with the distance between the anterior margin of the right ocular bulb and right interzygomatic line was 2.36 cm (Fig. 2). The lesion also pushed the right eye to the anterior.

Due to the possibility of destruction of the surrounding mass, the patient was scheduled for sclerotherapy instead of surgery. The patient then underwent 3 sclerotherapy sessions with interventional radiologists using bleomycin. After the first sclerotherapy session, there was still a bulging right eye with yellowish discharge and the patient was still unable to see. The patient then underwent the second sclerotherapy session in the next month, and the mass looked reduced in size, however, the patient was still unable to see.

**Fig. 3:**
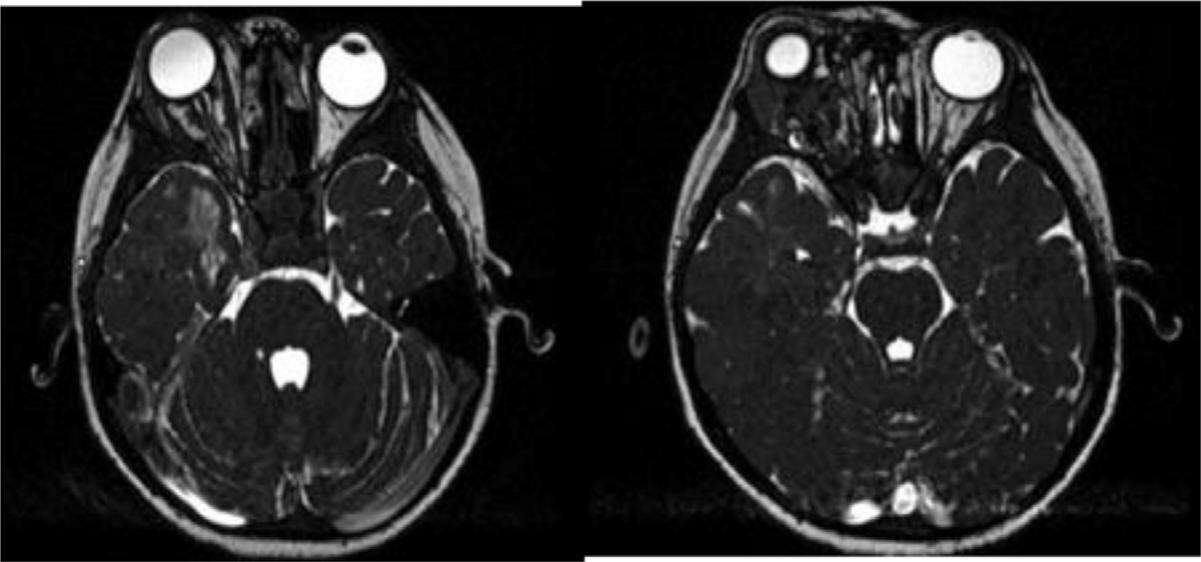
MRI postsclerotherapy showed a smaller inhomogeneous lesion in the right retrobulbar with the distance between the anterior margin of the right ocular bulb and the right interzygomatic line reduced to 2.12 cm.

**Fig. 4:**
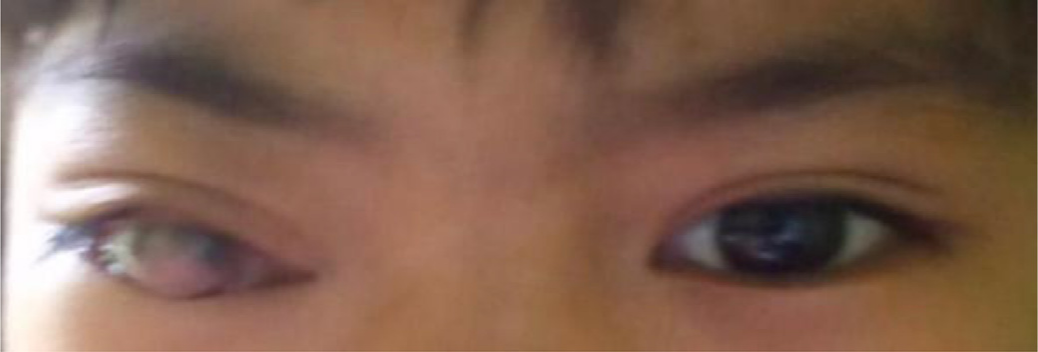
Clinical result after sclerotherapy

The third sclerotherapy session took place 1 month after the second one, and the proptosis was reduced significantly after the treatment (Fig. 4). A follow-up orbital MRI was performed to evaluate the result of sclerotherapy treatment, and it showed a reduced size of the mass, with the distance between the anterior margin of the right ocular bulb and the right interzygomatic line reduced to 2.12 cm (Fig. 3). However, the patient's vision was still impaired.

## Discussion

Lymphangioma is also known as lymphatic malformations. Lymphangioma is a multicystic, localized malformation that involves the lymphatic and vascular systems. The prevalence of lymphangioma has been estimated to be 1.1-5.3 cases per 10,000 live births. Lymphangioma accounts for 4% of all vascular tumors and roughly 25% of pediatric benign vascular tumors. Lymphangioma of the orbit is considered rare as it makes up about 1%-4% of all orbital lesions [6]. Orbital lymphangioma is divided into 4 major groups: (a) superficial lymphangioma, (b) deep lymphangioma, (c) combined lymphangioma, and (d) complex lymphangioma [1].

Ptosis and proptosis are the most common features of orbital lymphangioma, with the majority of cases appearing in the first decade of life. Traditionally, these lesions are not clinically recognized until bleeding into the lesion causes acute axial or nonaxial proptosis. This is common in infants as they become more active and prone to minor trauma. In the presence of an upper respiratory tract infection, lesions frequently appear acutely swollen. Occasionally, the lesion will cause progressive proptosis and ocular motility restriction. In rare cases, focal lesions may remain asymptomatic and be discovered by chance during neuroradiological imaging. Lesions can cause a mass effect, resulting in compressive optic neuropathy, most commonly after acute bleeding into the lesion. These potentially amblyogenic factors are significant findings because patients frequently present before full visual maturation, which may dictate the need for surgical intervention [1]. When assessing and managing patients with this lesion, the preservation of vision and prevention of amblyopia are top priorities.

Lymphangioma develops as a result of either congenital or acquired lymphatic system disorders. The congenital form is caused by an improper connection of the lymphatic ducts to the main lymphatic drainage duct and usually occurs before a patient reaches 5 years old. Acquired lymphangioma may develop from surgery, trauma, cancer, or radiation therapy. The failure of the lymphatic system to connect with or separate from the venous system, abnormal budding of lymphatic tissue from the cardinal veins, and obstruction of the efferent lymphatic vessels are causes of lymphangioma development. It can also be triggered by trauma, infection, chronic inflammation, and obstruction during embryonic development [7,8]. Histopathologically, superficial lymphangioma appears as a collection of large lymphatic pools in the subcutaneous tissue that communicate via dilated dermal lymphatic ducts lined with endothelial cells. Cystic or cavernous lymphangioma contains large, irregular vascular spaces lined with a single layer of flattened endothelial cells within a fibroblastic or collagenous stroma that may contain lymphocytes [9].

CT scans of a lymphangioma reveal a cyst-like mass with varying degrees of elevation and an increased cyst margin with bleeding. MRI reveals areas of acute and chronic bleeding within the mass and has been shown to identify feeder vessels that CT scans may not. The presence of such feeder vessels distinguishes this lesion from a lymphangioma. Angiographic studies show that the lesion is isolated from the arterial and venous circulations. Noninvasive dynamic MR angiography with contrast kinetics imaging, diffusion-weighted MRI, and ultrasonography have all been described as imaging modalities that can help differentiate lymphangioma from other vascular lesions. Lesions were found to branch into surrounding structures with all imaging modalities, causing image distortion without invading these structures [1]. Multiple cysts with high air-fluid levels are pathological findings. Radiology can help with the diagnosis of lymphangioma and assess the size of the lesion as well as the degree of involvement of surrounding structures, which may affect management planning. The cyst size also influences the type of intervention, with larger cysts, typically greater than 2 cm in diameter, being better candidates for sclerotherapy [10].

Lymphatic venous malformations are difficult to manage as they cause structural and functional disorders depending on their location, size, and relationship to adjacent tissues. Although conservative observation is often appropriate for asymptomatic lesions, because lymphatic venous malformations tend to develop with age, early intervention may be considered. Treatment for vision-threatening lesions will be more urgent. There were 2 types of treatment strategies: surgical and nonsurgical [6]. Nonsurgical treatment options include observation, sclerotherapy, and drug-based medical therapy. Observation of the lesion may be considered in patients who do not have any vision-threatening symptoms or severe physical disabilities. Medical therapy with medications such as PDE-5 inhibitors (Sildenafil) or mTOR inhibitors (Sirolimus) can be used. Other treatments for orbital lymphangioma include debulking and/or surgical excision; however, because the lesions are frequently adjacent to or intertwined with vital orbital structures, surgery is often difficult. Furthermore, the risk of recurrence is high. Despite the risks, surgical excision continues to be the mainstay of treatment for orbital lymphangioma [4,8,11].

Sclerotherapy is a preferred treatment method for cystic lesions, involving injection of agents with ultrasound guidance, resulting in scar formation and reduced cyst and lesion size. OK-432 (Picibanil), sodium tetradecyl sulfate, doxycycline, ethanol, pingyangmycin, and bleomycin are some of the specific agents used in orbital lymphangioma. The sclerosing effect is achieved by stimulating neutrophils and macrophages to produce cytokines such as IL-1, IL-2, IL-6, g-interferon, and TNF which increase endothelial permeability and activate neutrophils, macrophages, natural killer T cells, and lymphatic endothelial apoptosis, resulting in increased lymph fluid clearance and lesion resolution or shrinkage [6]. OK-432 (Picibanil) is a freeze-dried biological product prepared from the Streptococcus pyogenes strain (group A) by treatment with benzylpenicillin and heat. Proptosis and eyelid swelling improved gradually over 1 month and were completely resolved within 3 months of treatment using OK-432 [6,12]. The synthetic surfactant sodium tetradecyl sulfate (STS) is one of the sclerosing agents approved by the Food and Drug Administration (FDA). When this agent is injected intravenously, it causes intimal inflammation and thrombus formation, which usually occlude the injected vein. Following the formation of fibrous tissue, the vein is partially or completely obliterated, which may or may not be permanent [13,14]. Bleomycin is a cytostatic antineoplastic agent that inhibits the rapamycin (mTOR) pathway. These agents cause an endothelial-to-mesenchymal transition, which increases their scleroembolic effect [15]. In a study conducted in Bangladesh by Nuruddin et al, 12 cases of orbital lymphangioma were treated with intralesional injections of bleomycin at a dose of 0.5 mg/kg body weight (maximum 15 mg). Patients who require repeat therapy will be re-injected 4 weeks after their initial dose. As evidenced by radiology and digital photography, 50% of patients had complete resolution, and another 50% had resolution greater than 70% [11].

## Conclusion

Orbital lymphangioma is a rare multicystic lymphatic and vascular malformation that most commonly affects children in the head and neck region. Radiological imaging is important in confirming the diagnosis by identifying and evaluating the orbital mass. Orbital lymphangioma may be a challenging case to manage due to high risk of adjacent vital orbital structures destruction if surgery is performed. Sclerotherapy, as a less invasive intervention, is an effective alternative treatment for orbital lymphangioma with favorable result.

## Patient consent

Written informed consent for publication of their case was obtained from our patient's parent.

## References

[1] Saha K, Leatherbarrow B (2012). Orbital lymphangiomas: a review of management strategies. Curr Opin Ophthalmol.

[2] Woo YJ, Kim CY, Sgrignoli B, Yoon JS (2017). Orbital lymphangioma: characteristics and treatment outcomes of 12 cases. Korean J Ophthalmol.

[3] Raichura ND, Alam MS, Noronha VO, Mukherjee B (2017). A prospective study of the role of intralesional bleomycin in orbital lymphangioma. J AAPOS.

[4] Patel SR, Rosenberg JB, Barmettler A (2019). Interventions for orbital lymphangioma. Cochrane Database Syst Rev.

[5] Patel KC, Kalantzis G, El-Hindy N, Chang BY (2017). Sclerotherapy for orbital lymphangioma - case series and literature review. In Vivo.

[6] Nassiri N, Rootman J, Rootman DB, Goldberg RA (2015). Orbital lymphaticovenous malformations: current and future treatments. Surv Ophthalmol.

[7] Liu X, Cheng C, Chen K, Wu Y, Wu Z (2021). Recent progress in lymphangioma. Front Pediatr.

[8] Ha J, Yu Y-C, Lannigan F (2014). A review of the management of lymphangiomas. Curr Pediatr Rev.

[9] Bennet JM, Bhatt AR, Chau A, Desai SB (2020). Acute approach to orbital lymphatic malformations. Am J Interv Radiol.

[10] Khan SN, Sepahdari AR (2012). Orbital masses: CT and MRI of common vascular lesions, benign tumors, and malignancies. Saudi J Ophthalmol.

[11] Nuruddin M, Roy R, Mudhar S. Results of intralesional bleomycin sclerotherapy for treatment of orbital lymphangiomas at a tertiary eye care center in Bangladesh. 2019;4202:412–7. 10.1159/000495248 PMC6873038 31768364

[12] Fasching G, Dollinger C, Spendel S (2022). Treatment of lymphangiomas by means of sclerotherapy with OK-432 (Picibanil®) is safe and effective – a retrospective case series. Ann Med Surg.

[13] Das S, Agrawal A, Burathoki SK (2022). Orbital venolymphatic malformation treated with sodium tetradecyl sulfate: a case report. Cureus.

[14] Etezad M, Taher M, Hassanpoor N (2019). Sclerotherapy for eyelid and anterior orbital venous-lymphatic malformation. J Curr Ophthalmol [Internet].

[15] Nevesny F, Chevallier O, Falvo N, Guillen K, Malakhia A, Pellegrinelli J (2021). Bleomycin for percutaneous sclerotherapy of venous and lymphatic malformations: a retrospective study of safety, efficacy and mid-term outcomes in 26 patients. J Clin Med.

